# Toxic Determination of Cry11 Mutated Proteins Obtained Using Rational Design and Its Computational Analysis

**DOI:** 10.3390/ijms24109079

**Published:** 2023-05-22

**Authors:** Miguel O. Suárez-Barrera, Diego F. Herrera-Pineda, Paola Rondón-Villarreal, Efraín Hernando Pinzón-Reyes, Rodrigo Ochoa, Lydia Visser, Nohora Juliana Rueda-Forero

**Affiliations:** 1 Facultad de Ciencias Médicas y de la Salud, Instituto de Investigación Masira, Universidad de Santander, Bucaramanga 680003, Colombia; miguel.suarez@udes.edu.co (M.O.S.-B.); biomol.investigacion@udes.edu.co (D.F.H.-P.); diseno.molecular@udes.edu.co (P.R.-V.); ehpinzon@udes.edu.co (E.H.P.-R.); 2 Max Planck Tandem Group in Nanobioengineering, Institute of Chemistry, Faculty of Natural and Exacts Sciences, University of Antioquia, Medellin 050010, Colombia; 3 Centro de Bioinformática, Simulación y Modelado (CBSM), School of Bioinformatic, Universidad de Talca, Talca 3465548, Chile; 4 Biophysics of Tropical Diseases, Max Planck Tandem Group, University of Antioquia, Medellin 050010, Colombia; rodrigo.ochoa85@gmail.com; 5 Department of Pathology and Medical Biology, University of Groningen, University Medical Center Groningen, 9701 Groningen, The Netherlands; l.visser@umcg.nl

**Keywords:** *Bacillus thuringiensis*, Cry11, site-directed mutagenesis, *Aedes aegypti*, cancer cell lines

## Abstract

Cry11 proteins are toxic to *Aedes aegypti*, the vector of dengue, chikungunya, and Zika viruses. Cry11Aa and Cry11Bb are protoxins, which when activated present their active-toxin form in two fragments between 30 and 35 kDa respectively. Previous studies conducted with Cry11Aa and Cry11Bb genes using DNA shuffling generated variant 8, which presented a deletion in the first 73 amino acids and one at position 572 and 9 substitutions including L553F and L556W. In this study, variant 8 mutants were constructed using site-directed mutagenesis, resulting in conversion of phenylalanine (F) and tryptophan (W) to leucine (L) at positions 553 and 556, respectively, producing the mutants 8F553L, 8W556L, and 8F553L/8W556L. Additionally, two mutants, A92D and C157R, derived from Cry11Bb were also generated. The proteins were expressed in the non-crystal strain BMB171 of *Bacillus thuringiensis* and subjected to median-lethal concentration (LC_50_) tests on first-instar larvae of *A. aegypti*. LC_50_ analysis showed that the 8F553L, 8W556L, 8F553L/8W556L, and C157R variants lost their toxic activity (>500 ng·mL^−1^), whereas the A92D protein presented a loss of toxicity of 11.4 times that of Cry11Bb. Cytotoxicity assays performed using variant 8, 8W556L and the controls Cry11Aa, Cry11Bb, and Cry-negative BMB171 on the colorectal cancer cell line SW480 reported 30–50% of cellular viability except for BMB171. Molecular dynamic simulations performed to identify whether the mutations at positions 553 and 556 were related to the stability and rigidity of the functional tertiary structure (domain III) of the Cry11Aa protein and variant 8 showed the importance of these mutations in specific regions for the toxic activity of Cry11 against *A. aegypti.* This generates pertinent knowledge for the design of Cry11 proteins and their biotechnological applications in vector-borne disease control and cancer cell lines.

## 1. Introduction

The use of insecticidal proteins based on *Bacillus thuringiensis* (*Bt*) formulations is a promising approach to controlling different disease-transmitting vectors. Additionally, these toxins are ecosystem-friendly, offering an efficient and equally effective alternative to chemical treatments, and they do not present a risk to other living organisms, including humans. However, the resistance acquired by insects against Cry toxins is becoming a challenge [1]. Hence, current research has been focused on the development of more robust and stable Cry toxins to better control vector-borne diseases.

Cry11 proteins have three active domains that act against vector-borne diseases in the Dipteran order [2]. The best-characterized member of this family is Cry11Aa, a protein of approximately 70 kDa produced by *Bt* subsp. *israelensis* (*Bti*), in which proteolytic activation removes approximately 28 amino acids from the N-terminus. The resulting protein is cleaved into two fragments of 32 and 34 kDa. These fragments remain associated and exhibit insecticidal activity against *A. aegypti* [3,4]. Cry11Bb, produced by *Bt* subsp. *Medellín* (*Btmed*) [2] has the distinction of having toxic effects on mosquito genera such as *Culex* and *Anopheles*. This toxin weighs 94 kDa, and its toxic effects result from solubilization and proteolytic processing into fragments of 30 and 35 kDa in the midguts of insects [5].

Protein engineering has emerged as a tool for developing modified Cry proteins with novel activities. Directed evolution methodologies focus on amino acid substitution and the insertion or elimination of DNA sequences [6]. For the improvement of Cry toxins, strategies related to rational approaches such as protein engineering [7], domain exchanges [8], use of synthetic peptides involved in the enhancement of protein stability [9], and site-directed mutagenesis [10], as well as non-rational approaches such as DNA shuffling, error-prone PCR, and microarrays with phage libraries have also been used [7]. These approaches, in addition to obtaining improved variants, have allowed the identification of insect-dependent toxin activation mechanisms, stability, oligomerization, and the modulation of interactions with membrane receptors in the epithelial cells of the midgut of insects [11]. Simultaneously, mutagenesis studies have enabled the development and/or conditioning of new molecular biology techniques, providing alternatives to overcome the resistance acquired by insects and the development of green biotechnology that could reduce the negative environmental impact [12].

It has been reported that small changes in amino acid residues of the three-domain Cry proteins provided an improvement in their toxicity or target changes [7]; for example, the change of a residue (V171C) in the δ-Cry1Ab endotoxin showed a loss of toxic activity against *Manduca sexta*, an insect susceptible to this toxin, while increasing its toxicity (25-fold) against *Lymandria dispar*, since this modification probably contributed to greater protein stability and a better binding rate to the midgut receptors [13]. Additionally, some studies have reported genetic improvements in Cry11 using DNA-shuffling techniques or site-directed mutagenesis [7]. Our group is focused on the directed evolution of Cry11 and Parasporin proteins through DNA shuffling [10], thermodynamics and secondary structure formation analysis [14], and the development of an in silico/in vitro workflow [15]. Previous reports highlighted that variant 8 obtained via DNA shuffling from Cry11Aa and Cry11Bb exhibited up to 3.78- and 6.09-fold higher toxicity to *A. aegypti* than Cry11Aa and Cry11Bb, respectively [10]. Mutations in variant 8 are responsible for the increase in toxicity; however, it is important to assess the role of each mutation to understand the mechanism of action of Cry11. Simultaneously, Cry proteins have been described as possible adjuvants because of their resistance and stability in highly alkaline environments during the development of vaccines [16], making these bioproducts relevant not only in environmental applications but also in the biomedical field. Our group is currently focused on generating new proteins with anticancer activity, considering the possibilities with the mutations obtained with variant 8 and subsequent proteins. We also assessed whether mutations in these proteins influence the anticancer activities. Therefore, molecular analysis and determination of toxicity were performed using *A. aegypti* larvae with the colorectal cancer cell line SW480 as targets. Mutants 8F553L, 8W556L, and 8F553L/8W556L were used to analyze mutations in variant 8 at these positions. Furthermore, the A92D and C157R variants were used to analyze mutations at residues 92 and 157 in the Cry11Bb toxin. Finally, a computational analysis of the possible implications of the mutations at positions 553 and 556 in the three-dimensional structure of the Cry11Bb toxin was performed.

The results presented here elucidate the role of induced mutations in proteins with insecticidal and anticancer activities. This work provides new insights into the structure–function relationship of the parasporal proteins of *Bt* and their applications as insecticidal toxins and anticancer proteins.

## 2. Results

### 2.1. Variants Were Successfully Obtained from Variant 8 and Cry11Aa

The designed primers were effective in obtaining each variant. The constructs pSV2-L553F, pSV2-L556W, and pSV2-L553F/L556W were verified using a restriction enzyme assay with *HindIII* and *SacI*, and were observed in an agarose gel; a double band was obtained, of which one of 5 kb corresponds to the size of the plasmid and the other one of approximately 2 kb corresponds to the size of the Cry gene (Figure S1). Simultaneously, the constructs pSV2-A92D and pSV2-C157R showed a linearized vector pSV2 digested with *HindIII* and *SacI* (5 kb) and bands of the inserts between 2 and 3 kb. Each construct was used to transform the crystalline strain BMB171; the transformation efficiency was established at ≈10^9^ CFU·µg^−1^ of the DNA (construct). Sequencing of these constructs revealed that the individual mutants, L553F and L556W, were successfully reversed from cytosine to adenine and from guanine to thymidine, respectively.

### 2.2. Cry 11 Proteins and Variants Are Produced in Bt BMB171

The viable cell count for each mutant and control was >1 × 10^9^ CFU·mL^−1^. Dry weight estimates ranged from 0.023 to 0.061 g·mL^−1^. The protein concentration obtained was between 12.32 and 40.74 mg·mL^−1^ as measured using the Bradford method.

The electrophoretic protein pattern was verified using 10% SDS-PAGE. The samples stained with Coomassie R-250 blue showed a protein pattern with a recognizable band close to 70 kDa for the L553F, L556W, L553F/L556W, and Cry11Aa proteins, whereas for the mutants A92D, C157R, and Cry11Bb, the observed band was close to 90 kDa. For the specific case of BMB171, the banding of the Cry11 protein was not shown. After activation with proteinase K, a double band was observed for each of the mutant and native proteins, with molecular weights of 30 and 35 kDa, confirming the activation of the protoxin to the toxin (Figure S2).

### 2.3. The Residues Phenylalanine (553) and Tryptophan (556) Are Relevant to the Insecticidal Activity of the Variant 8 Protein

Variant 8, obtained via DNA shuffling, presented insecticidal activity approximately five times higher than that of the parental genes Cry11Aa and Cry11Bb. By reversing the mutations in residues 553, 556, and 553/556, no toxic activity was detected with an LC_50_ of up to 500 ng·mL^−1^ (Table 1), indicating the importance of these residues (F and W) for the toxicity of this protein. Similarly, no toxic activity was detected for C157R; the mutant A92D showed a decrease in the activity of 11.44 compared with Cry11Bb.

### 2.4. Cry Proteins Have an Antiproliferative Effect on the SW480 Cell Line

**Figure 1:**
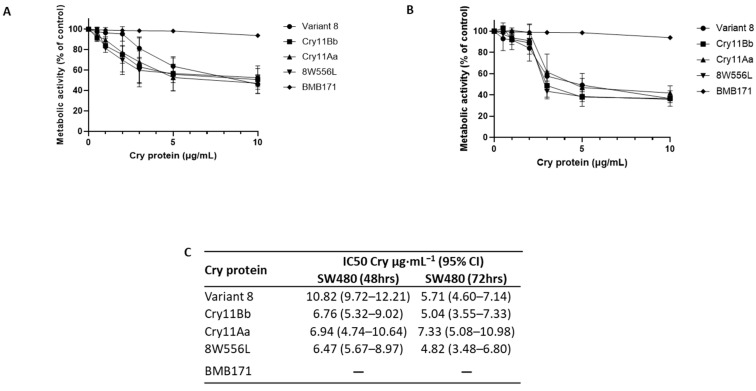
Cytotoxicity activity of the Cry proteins on the colorectal cancer cell line SW480 at 48 h (**A**) and 72 h (**B**). The measurements were determined with Alamar Blue staining. The IC_50_ was calculated with different concentrations of Cry proteins (0.5–10 µg·mL^−1^) at 48 and 72 h (**C**). No IC_50_ was calculated for BMB171.

We used strain BMB171 as a Cry-expressing negative control, which showed no detectable or slight activity against SW480 cells. The activities of all Cry proteins, including the controls Cry11Aa and Cry11Bb, were similar, measured at 10 µg·mL^−1^ for 48 and 72 h, with metabolic activities of 50% and 30%, respectively (Figure 1A,B). Furthermore, the calculated IC_50_ showed no differences between the controls Cry11Aa and Cry11Bb, and 8W556L, with an IC_50_ of 6.5–7.0 µg·mL^−1^. Surprisingly, 8W556L was 1.6 times more active for this cell line than Variant 8. This suggests that residue 556 might be relevant for anticancer activity.

### 2.5. Modeled Structures by De Novo Methodology

**Figure 2:**
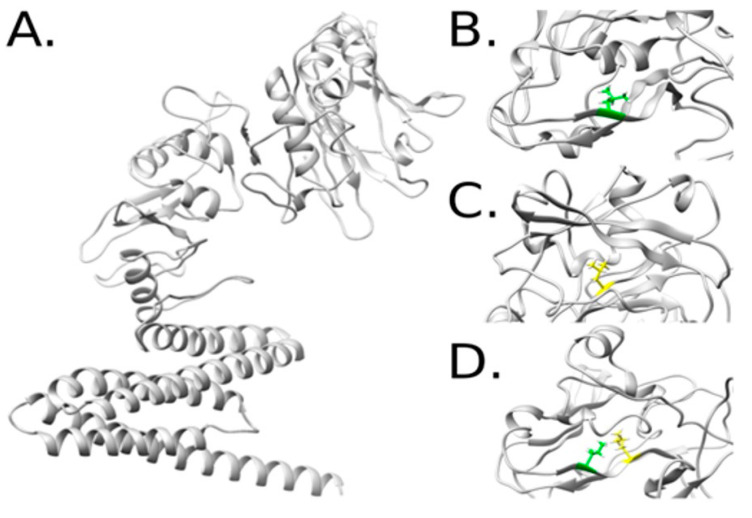
Structural model of variant 8 (**A**). Representations of the variants 8F553L (**B**), 8W556L (**C**), and the combination of 8F553L and 8W556L (**D**) after running MD simulations of the mutated complexes.

Owing to the lack of available homologous structures for the designed variant 8, de novo methodology was used to obtain energetically favorable models for further analysis. The highest identity percentage for Cry11Bb was 25% for related structures reported in the Protein Data Bank (PDB) [17] and similar results were obtained for the variant 8 protein sequence. Following de novo contact-based modeling, a structural view of variant 8 and the mutants 8F553L, 8W556L, and 8F553L/8W556L is shown in Figure 2.

**Figure 3:**
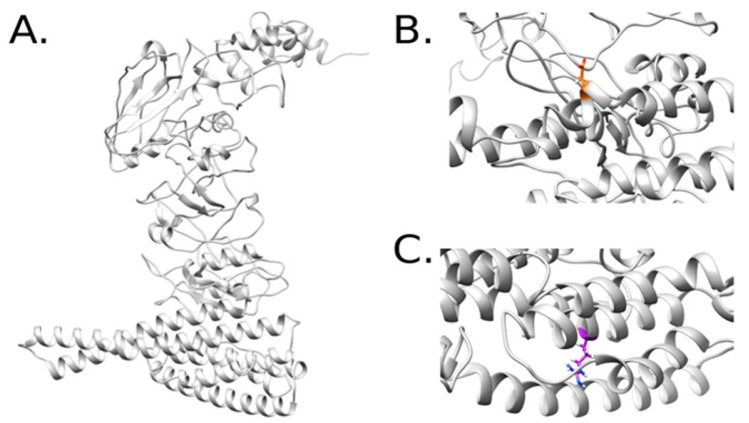
Structural model of Cry11Bb (**A**). Representations of the variants A92D (**B**) and C157R (**C**) after running MD simulations of the mutated complexes.

The structure of variant 8 was modeled based on a sequence of 571 amino acids, which were initially subjected to other modeling methods with unsuccessful results. The selected mutations were located in a domain with non-representative matches to other proteins with available structures. However, this region still folds into a representative backbone that confers stability and is crucial for subsequent interactions with other molecular entities. A similar analysis was performed after modeling de novo Cry11Bb and sampling its variants for convergence purposes (Figure 3).

For this isoform, the variants are located in a region composed of multiple helices but pointing in opposite directions from a group of helices (Figure 3B,C). This set of models was more challenging to predict owing to the larger sequence size (750 amino acids). However, with the proposed method and energy evaluations, we conclude that the models are suitable for additional computational analysis.

Molecular dynamics simulations were performed with an implicit solvent in triplicate for domain II of variant 8 and Cry11 wild-type molecules, where amino acid mutations were performed based on the results, trajectory, root-mean-square deviation of atomic position (RMSD), root-mean-square fluctuation (RMSF), and radius of gyration.

**Figure 4:**
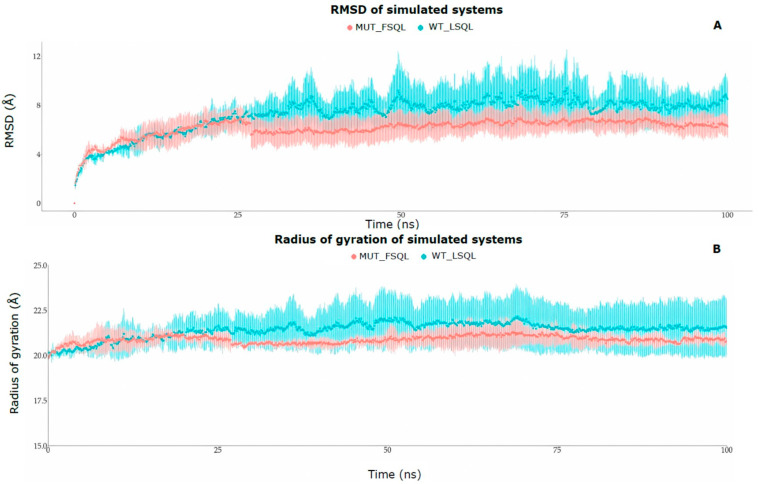
RMSD of simulated (**A**): red color (variant 8) and blue color (Cry11). The radius of gyration of the simulated (**B**): red color (variant 8) and blue color (Cry11).

RMSD analysis (Figure 4A) showed that the mutant molecule (variant 8) registered greater stability when exposed to solvent than the wild-type molecule (Cry11). This stability can be attributed as a consequence of the two mutations made at positions 553 and 556; in the same way, the greater stability of the mutant molecule seems to favor the interaction of the molecule in the toxicity process.

The radius of gyration analysis (Figure 4B) shows that the wild-type protein (Cry11) retains a less rigid structure after the first quarter of the DM simulation has elapsed, while the mutated protein, although it starts with a less rigid structure, is presented in the course of the rest of the interaction to the more compact solvent. This preferentially compact conformation of the mutant after a small non-rigid interval seems to favor the interaction of the molecule in the process that desiccates the toxicity of the mutant. The RMSD and radius of gyration calculations between the systems showed a statistically significant difference in the *t*-test, with a *p*-value of 0.000 (<0.05).

RMSF analysis allowed us to observe some amino acids that presented average differences greater than 2 Å (Table 2) between the wild and mutant systems; the amino acids with the greatest difference could explain the increase in the toxicity of the mutant. Some amino acids of interest present differences greater than 3 Å in the mutant: LEU59, SER70, LEU87, and TRP265; the latter shows the greatest difference of 5.78 Å and corresponds to one of the mutated amino acids (Table 2). These amino acids that significantly change their spatial conformation with respect to their initial positions in the wild-type protein could be determining factors in the increase in the toxicity of the mutant protein. It is highlighted that the amino acid LEU556, one of the mutated amino acids, has a very high conformational difference.

## 3. Discussion

The efficiency of Cry proteins produced by *Bt* is high, and only small amounts are generally necessary to cause toxicity [7]. Over time, the target insects for these proteins have developed various resistance mechanisms associated with the alteration of intestinal proteins that activate Cry proteins, modification of receptor-binding sites in the midgut, and synthesis of oligosaccharides, among others [18,19,20]. Therefore, there is a need to apply various genetic improvement strategies such as DNA shuffling, domain swapping, and/or truncation [7]. These approaches will aid in controlling resistant mosquito populations and in elucidating the structure, function, and mechanisms of action of these proteins [11].

In the present study, a site-directed mutagenesis approach was used to explore the role of positions 553 and 556 in the differential activity of variant 8, a mutant obtained via DNA shuffling of Cry11Aa and Cry11Bb [10]. Herein, we present a molecular analysis of the lethality of these mutated variants in *A. aegypti* larvae. The LC_50_ values obtained for the mutants were >500 ng·mL^−1^, suggesting that the reversal of amino acids located at positions 553 and 556 directly affected the lethality of these variants and that the chemical nature of these residues probably plays a significant role in their structural function. Additionally, the 556 variant was tested in SW-480 colon cancer cell lines, as Cry proteins that lack insecticidal activity could be candidates for anticancer proteins. The results indicated that the insecticidal activity of 8W556L was null, but when tested against SW480, its cytotoxic activity was almost two times higher than that of variant 8 at 48 h and similar to the cytotoxic activity of Cry11Aa and Cry11Bb. At 72 h, the activities of all Cry proteins were similar. This difference in activity against both targets indicates that the mechanism of action probably depends on the composition of the cell membrane [20,21,22], and the mutation of 8W556L is located in domain III; this region is involved in the processes of oligomerization and stabilization of the Cry proteins [3,21].

It is not the first time that Cry proteins or peptides obtained from Cry proteins have been used to control cancer cell lines. In 2022, it was reported that the co-administration of inactivated and activated forms of Cry1Ac with doxorubicin in a triple-negative breast cancer mouse model enhanced tumor immunity [22]. In 2021, Rendon-Marin et al. evaluated the activity of peptides obtained from Cry11Bb and found that the peptide BTM-P1 had a toxic effect on Caco-2 and MCF-7 cells, inducing apoptosis in MCF-7 cells with low hemolytic activity [23]. The background of the reported information and the study presented in this paper make exploring new targets for Cry proteins a promising approach for biotechnology and human medicine.

Computational simulations of molecular dynamics allowed us to establish hypotheses about the influence of the amino acids at positions 553 and 556 (mutants 8F553L, 8W556L, and 8L553F/8 L556W) on the structural behavior of domain II arranged in a solvent for the Cry11 and variant 8 proteins. Molecular simulation studies demonstrated that the structure of domain II in the mutated variant 8 protein remained with greater energy stability, showing a compact structure throughout the simulation time; 3% of the amino acids in the mutated domain changed their initial configuration at a distance greater than 2 Å, at the positions LEU350, SER361, LEU378, and LEU556. Amino acids such as phenylalanine and tryptophan have chemical characteristics that differ from leucine. For leucine, its conformation is given by aliphatic side chains, whereas [24,25] phenylalanine and tryptophan are made up of aromatic rings [24] that are involved in hydrophobic interactions and tend to be positioned at the center of a protein, excluded from the solvent, allowing better stability and function. Electron delocalization also plays an important role. These results suggest that the indolyl NH functional group in the tryptophan residue may be associated with protein–receptor interactions. Previous studies on chemical modifications of tryptophan, such as sulfenylation, led to a loss of toxicity in mice and apparent binding affinity for rat brain and cockroach synaptosomal preparations [24]. Currently, different scientific fields are exploring the role of tryptophan in the photon-sensing capabilities of living organisms through the resonance delocalization of π-electrons [25].

Cry proteins are highly conserved within their three domains; any changes in any residues either enhance activity, induce loss of activity, or change the target, depending on the domain affected. Studies conducted by Fernandez et al. [3] showed that the toxicity of Cry11Aa is affected by amino acid substitutions via site-directed mutagenesis of hydrophobic residues (alanine) to hydrophilic residues, such as glutamic acid. The loops responsible for binding to membrane receptors have a greater binding affinity as they are hydrophobic regions; their chemical properties continue to be preserved because they are not exposed to the solvent. Similarly, in studies based on loop 3 of the Cry1Ab protein where a substitution of phenylalanine for alanine was made, a significant reduction in the toxic activity (3.5 times) towards insects of the order Lepidoptera was observed since binding to the receptors present in the insect membrane did not occur because alanine, having an aliphatic chain within its chemical characteristics, hinders the generation of hydrogen bonds between the protein and the receptor [26]. This result is different from others because phenylalanine is an amino acid with aromatic chemical characteristics. In contrast, Tiewsiri and Angsuthanasombat reported a high decrease in toxicity against *A. aegypti* when making alanine substitutions in the positions where aromatic amino acids (tryptophan, tyrosine, and phenylalanine) were found at residues 243, 249, and 264, respectively, located in the α-7 helix, which confirmed that aromatic residues are involved in the increase or decrease in toxicity [27].

The receptor binding capacity of Cry toxins is attributed primarily to domain II rather than domain III, although the latter may be involved in the binding processes [28]. Several studies have shown that domain III of various Cry toxins is involved in receptor binding [28,29]. For example, the substitution of domain III in Cry1Ab and Cry1Ac with domain III of Cry1Ca resulted in an approximately 10-fold increase in toxicity towards *Spodoptera exigua* and the recognition of receptor proteins present in insect membranes [30]. Moreover, Mushtaq et al. designed a hybrid toxin that shared domains I and II of Cry1Ac (active sites for *Anticarsia gemmatalis* and *Chrysodeixis includens*) and domain III of Cry2Ac7 to generate a new protein with increased toxicity against both species [31]. The results indicated that the toxicity against *A. gemmatalis* and the binding to its membrane receptors were maintained; a different case occurred with *C. includens,* where no toxicity was found [31].

The A92D and C157R variants obtained by site-directed mutagenesis of Cry11Bb were intended to demonstrate the significance of the amino acid residues located at positions 92 and 157 in domain I. The results obtained for the LC_50_ assays against *A. aegypti* showed a 13- and 21.8-fold decrease in toxicity for the A92D and C157R variants, respectively, compared with the parental Cry11Bb (Table 1). Genetic manipulation of domain I is mainly focused on studying or improving the toxic activity of Cry proteins against the formation of pores or ion channels in the midgut membranes of insects. This domain comprises various polar amino acids that directly participate in the formation of hydrogen bonds or salt bridges [7,32]. Wu and Aronson reported that the substitution of specific residues at position A92D of the Cry1Ab protein caused a reduction in toxic activity due to the substitution of the negatively charged amino acid residues at this position, which caused detrimental effects on pore formation in *M. sexta* [33]. Additionally, Álzate in 2010 reported a three-fold decrease in the insecticidal activity of the Cry1Ab toxin against *M. sexta* when modifying the L157R position, which correlated with the degree of loss of transport activity of ions generated by the chemical nature of the modified amino acids [13].

This work is promising for the study of the structure–function relationship of Cry11 proteins as a valuable biotechnological asset that, in the future, might be an alternative to the use of chemical insecticides in the control of mosquitoes, and may aid in preventing the spread of vector-borne diseases, and simultaneously, in the collecting of information to enhance modeling and in the obtaining of biomolecules directed to the control of mosquito-borne diseases considered a problem of public health worldwide [34]. This work also explored the potential biomedical applications of this parasporal protein in controlling the proliferation of primary adenocarcinomas. Further research is required to elucidate whether these mutations broaden the targets of these proteins while contributing to a deeper understanding of the structure–function relationship in the Cry11 protein family.

## 4. Materials and Methods

### 4.1. Culture Media, Strains, and DNA Extraction

The bacterial strains were cultured in Luria Bertani (LB) broth supplemented with ampicillin 50 µg·mL^−1^ for the transformants of *Escherichia coli* DE3BL21 strain and chloramphenicol 10 µg/mL for the transformants of the non-crystal producing *Bt* BMB171 strain; Plasmid DNA was extracted using the Wizard Minipreps kit (Promega^®^, Madison, WI, USA) following the manufacturer’s instructions. Agarose gel electrophoresis was performed at a concentration of 0.8% and stained with SYBR-safe 10×.

### 4.2. Synthesis and Design of Cry11Bb and Variant 8 Mutants

#### 4.2.1. Cry11Bb Mutant Library

Variants obtained using rational approximations for Cry11Bb were designed based on the toxin–receptor interaction analyzed in previous studies [7,13,33,35,36]; the Cry11Bb sequence was modified at position 92, changing an alanine for aspartic acid (A92D), and in the second variant, changing the cysteine for arginine at position 157 (C157R). The genes were synthesized at Genescript (Piscataway, NJ, USA) and cloned into the expression vector pSV2, followed by electroporation in *E. coli* DE3BL21. The transformation was verified using plasmid extraction, followed by a restriction assay using *HindIII/SacI* (NEB, Manchester UK), and then visualized using agarose gel electrophoresis.

#### 4.2.2. Variant 8 Mutant Library

Primers were designed using the web service https://www.lifetechnologies.com/order/oligoDesigner/mutagenesis (accessed on 8 January 2021). The pSV2 plasmid harboring the variant 8 gene was used as the template; the selected positions for mutations were 553, 556, and a double mutation 553 and 556. The primers used are listed in Table S1 (Supplementary Material). The GeneArt™ Site-Directed Mutagenesis System kit (Thermo Fisher Scientific, Waltham, MA, USA) was used for amplification, following the manufacturer’s instructions. The constructs were amplified, transformed into chemically competent *E. coli* TOP10 cells, and electroporated into *Bt* BMB171 cells. The library was verified via sequencing the constructs; for this, 20 μg·μL^−1^ were sent to Macrogen (Seoul, Republic of Korea) and sequenced using the oligos: 8MDF (CTGCTGGAATACGGGTACA) and 8MDR (AATTTCGGTTGTTCAATAAGT).

### 4.3. Obtaining Final Complete Culture (FCC) of Bt

Mutants L553F, L556W, L553F-L556W, A92D, and C157R and recombinants expressing Cry11Aa and Cry11Bb toxins, together with the non-crystal-forming strain *Bt* BMB171, were grown in 10 mL of LB broth with chloramphenicol at 10 µg·mL^−1^ and incubated at 30 °C with shaking at 300 rpm for 7 d. After 48 h of incubation, spore and crystal formation were examined using malachite green staining. To obtain FCC, 5 mL of each culture was centrifuged at 10,000 rpm for 5 min at 4 °C. The supernatant was discarded, and 1 mL of 1M NaCl was added. The preparation was incubated for 1 h at 30 °C with stirring at 50 rpm. Subsequently, it was centrifuged at 12,000× *g* for 5 min at 4 °C. The supernatant was discarded, 1 mL of 1× phosphate buffered saline (PBS) was added, and the pellet was homogenized and centrifuged under the above-mentioned conditions. This procedure was repeated twice, and cells were frozen in 1 mL of PBS.

### 4.4. Protein Electrophoresis on Sodium Dodecyl Sulfate-Polyacrylamide Gel Electrophoresis (SDS-PAGE)

The samples were run on a 10% acrylamide gel. The gel and electrophoretic run were prepared following the instructions in the protocol book by Sambrook [37]. Coomassie blue R-250 and destaining solution were used for gel development for 30 min. The samples were treated with proteinase K at 10 μg·mL^−1^ to verify the characteristic activated fragments of Cry11 proteins.

### 4.5. Protein Preparation and Half-Lethal Concentration (LC_50_)

The cultures of each mutant dissolved in PBS 1× were subjected to heat stress, which was performed as follows: 100 µL of each culture was incubated at 72 °C for 20 min followed by incubation at 4 °C for 10 min. Dilutions of 10^−1^ to 10^−5^ were made with a final volume of 100 µL, and then the dilutions were plated on LB agar with chloramphenicol (10 µg·mL^−1^) and incubated at 30 °C for 24 h. The results were reported in spores/mL of culture. Proteins were solubilized by taking 200 µL of the final culture of each mutant and parental strain and adding 800 µL of crystal solubilization buffer (50 mM NaOH and 10 mM EDTA; pH 11.7). This solution was incubated overnight at 4 °C, followed by centrifugation at 27,000× *g* for 5 min at 4 °C. The supernatant was collected, and the volume was adjusted to 1 mL with 0.1 M Tris-base pH 7.4. To quantify the proteins, the Bradford micro-technique was used at 540 nm with a NanoDrop 2000. Three measurements were performed for each variant.

To establish the amount corresponding to the total protein of the cell mass, a dry weight estimation was performed by taking 500 µL of the FCC of each variant and centrifuging at 15,000 rpm at 4 °C for 30 min. The supernatant was discarded, and the pellet dried for 48 h at 20 °C. Subsequently, dry weight measurements were performed. Lethality tests were performed on each of five larvae of *A. aegypti* in instar I in triplicate with three replicates. To estimate lethality, live larvae were counted and translated into the percentage of dead larvae. Statistical analyses were performed using the ProBit function, and the mean lethal concentration (LC_50_) was calculated using R-Project Software v4.2.0. [38].

### 4.6. Variants of Cry in the Control of the Human Colorectal Cancer Cells

The solubilized proteins were activated using proteinase K (185 μg·mL^−1^) for 1 h at 37 °C. Phenylmethylsulphonyl fluoride was added (final concentration: 1 mM) to inhibit the proteolytic activity. SDS-PAGE was performed to confirm the presence of Cry proteins. Protein concentration was determined using the Bio-Rad Protein Assay (Bio-Rad Laboratories, Mississauga, ON, Canada).

Colorectal cancer cells (20,000/well) of SW480 (obtained from American Type Culture Collection-ATCC-Accession number CCL-228) were plated, and RPMI culture medium (Sigma Aldrich^®^, St. Louis, CO, USA) supplemented with 10% fetal bovine serum was added to make a final volume of 100 µL. Subsequently, 100 µL of culture medium with activated proteins at twice the concentration to be evaluated (0.5, 1, 2, 3, 5, and 10 µg/mL) were added and incubated for 48 and 72 h at 37 °C. Finally, 10 µL of Alamar blue was added to each well and incubated for 4–6 h to measure the fluorescence (Ex 560 nm and Em 590 nm) and thus determine the IC50. The results are expressed in terms of metabolic activity using the following formula:

(1) $$\%\;metabolic\;activity=\frac{cell\;absorbance\;with\;treatment}{cell\;absorbance\;without\;treatment\;}\;\times \;100$$

### 4.7. Structural Modeling of Cry11 Variants

To design the Cry11 mutants, a protocol was prepared to model two Cry11 variant structures: variant 8 and Cry11Bb. To fit the model, we applied a de novo prediction method called trRosetta [39], which was built using direct energy minimization with Rosetta Commons functionalities [40], including inter-residue distances and orientation restraints predicted by a deep neural network (https://yanglab.nankai.edu.cn/trRosetta/), accessed on 10 April 2022.

The best model per sequence was subjected to energy and geometrical evaluations using the ProSa web server (https://prosa.services.came.sbg.ac.at/prosa.php) accessed on 15 April 2022 and the structural assessment pipeline available on the SwissModel portal (https://swissmodel.expasy.org/assess) accessed on 15 April 2022.

### 4.8. Generation of Additional Variants and Sampling of the Models

Based on the initial models, a subsequent set of variants was predicted by performing single-point mutations on the variant 8 and Cry11Bb structures. The first variation consisted of mutations at positions 553 and 556 (mutants F553L, W556L, and F553L/W556L) of the variant 8 model. Single-point mutations were performed using UCSF Chimera [41] through the prediction of rotamers using Dunbrack backbone-dependent libraries [42], in which the Cry11Bb structure was used to generate the variants A92D and C157R. Molecular Dynamics (MD) was performed in triplicate for Cry11 and variant 8 in periods of 100 ns. The simulation protocol for all systems was solvated with TIP3P63 water molecules with a margin of at least 14 Å from the protein, and Na^+^ and Cl^−^ ions were added to ensure the neutrality of the system. At an ion concentration of 150 mM, the Amber ff1464 and GAFF65 force fields for the proteins were implemented for all systems. The system’s energy was first minimized and balanced using Amber18.67 software for complexes, and stepped minimization, heating, and balancing were performed. Complexes were first minimized for 1000 conjugate gradient steps, followed by 10,000 unrestricted steps, heated in 200 ps unrestricted steps, and then in an NPT set (7 ns) up to 300 K. The protocol for the setup of the protein system simulations on the GPU was followed. Finally, simulations were performed under NPT conditions (Langevin thermostat with Berendsen Barostat) to ensure the balance of the entire system.

## Figures and Tables

**Table 1:** LC_50_ for each of the variants obtained in this study.

Origin of *Bt* Protein	Strain	LC_50_ (ng·mL^−1^) (95% Cl)
		*Aedes aegypti*
Variant8 Derivatives	8F553L	>500
	8W556L	>500
	8F553L/8W556L	>500
Cry11Bb Derivatives	C157R	>500
	A92D	313.71 (158.73–317.46)
Controls	Cry11Aa	39.20 (20.04–45.12)
	Cry11Bb	27.40 (15.64–31.47)
	Variant 8	8.22 (2.01–9.33)

**Table 2:** The difference in RMSF is the difference in Å of the spatial position of the same amino acid in the two systems, wild-type (Cry11), and mutant (Variant 8).

Amino Acids	Position	The Difference in RMSF between the Two Systems (Å)
LEU	350	3.71 Å
SER	361	3.72 Å
LEU	378	3.84 Å
PRO	512	2.40 Å
PHE	524	2.18 Å
THR	534	2.00 Å
LEU	556	5.78 Å
SER	562	2.32 Å

## Data Availability

The data presented in this study are available on request from the corresponding author. The data are not publicly available due to privacy agreements between the parties.

## Supplementary Materials

The following supporting information can be downloaded at: https://www.mdpi.com/article/10.3390/ijms24109079/s1.
