# Supplementary material for: Toxic Determination of Cry11 Mutated Proteins Obtained Using Rational Design and Its Computational Analysis
Source: Int J Mol Sci. 2023 May 22;24(10):9079. doi: 10.3390/ijms24109079 (PMC10219489; doi:10.3390/ijms24109079)
Supplement: Supplementary file 1 [file ijms-24-09079-s001.zip › ijms-2375424-supplementary.pdf]

International Journal of *Molecular Sciences*

Article

Toxic determination of Cry11 mutated proteins obtained using rational design and its computational analysis

Suarez-Barrera M.O[1-3], Herrera-Pineda Diego[1], Rondón-Villarreal P[1], Pinzón-Reyes EH[1,4], Ochoa Rodrigo[5], Visser L[1], and Rueda N.J[1]*

[1] Facultad de Ciencias Médicas y de la Salud, Instituto de Investigación Masira, Universidad de Santander
[2] Department of Pathology and Medical Biology, University of Groningen, University Medical Center Groningen, Groningen, The Netherlands
[3] Max Planck Tandem Group in Nanobioengineering, Institute of Chemistry, Faculty of Natural and Exacts sciences, University of Antioquia.
[4] Centro de Bioinformática, Simulación y Modelado (CBSM), School of Bioinformatic, Universidad de Talca, Talca, Chile
[5] Biophysics of Tropical Diseases, Max Planck Tandem Group, University of Antioquia, 050010 Medellin, Colombia
* Correspondence: Juliana.forero@udes.edu.co

Supplementary materials

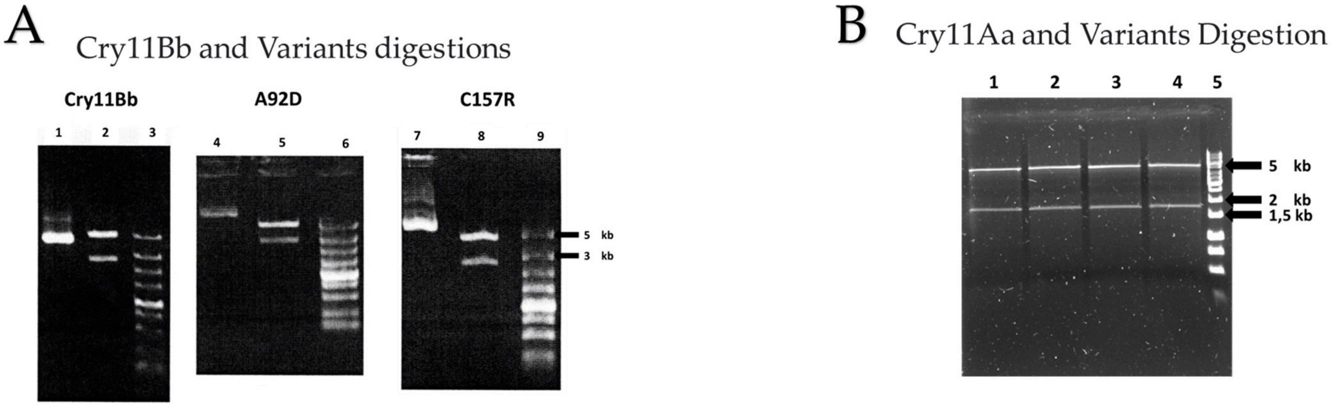

Figure S1. Restriction enzyme assay HindIII/ SacI for every native and variant gene obtained. 1) 553; 2) 556; 3) 553-556; 4) Cry11Aa; 5) Weight marker.

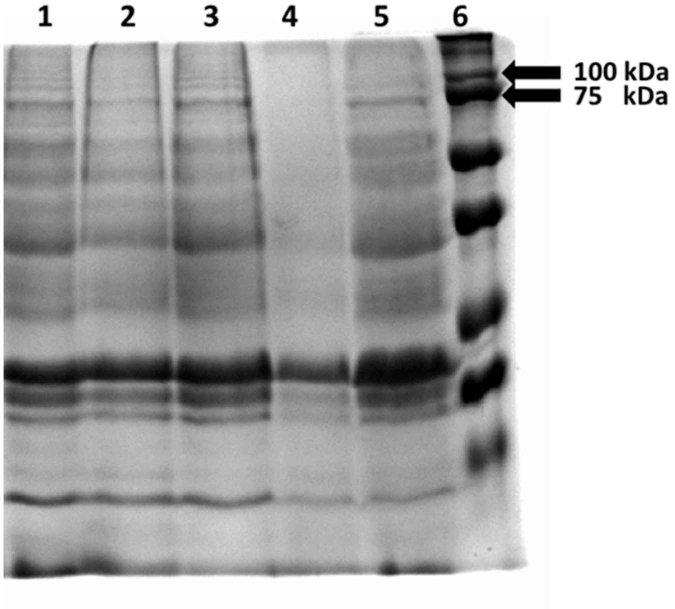

Figure S2. A. SDS-PAGE Cry11Aa – 72 kDa. Lane 1. Variant 553; Lane 2. variant 556; Lane 3. Double mutant 553-556; Lane 4. Negative Control BMB171; Lane 5. Positive Control Cry11Aa; Lane 6. Weight marker.

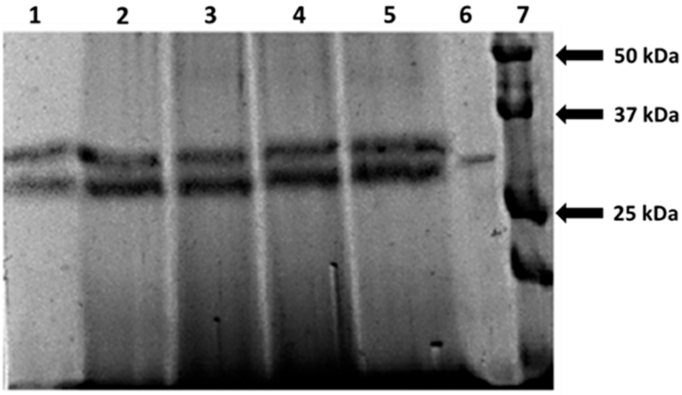

Figure S2. B. SDS-PAGE Cry11Aa active toxin (32- 34 kDa). Lane 1. Cry11Aa; Lane 2. Variant 8Aa; Lane 3. Variant 553; Lane 4. Variant 556; Lane 5. Variant 553-556; Lane 6. Negative control BMB171; 7) Weight marker.

Table S1. Oligos designed to obtain the variants 8Cry11Aa-553, 8Cry11Aa-556 and 8Cry11Aa-553/556.

Position	ID	Sequence
553	3C8F	5′-AGAAGAGTGGTATTTATCGCAGTGGTTTGTA-3′
	3C8R	5′-TACAAACCACTGCGATAAATACCACTCTTCT-3′
556	6C8F	5′-GGTATTTCTCGCAGTTGTTTGTAGTAAAAGA-3′
	6C8R	5′-TCTTTTACTACAAACAACTGCGAGAAATACC-3′
553-556	36C8F	5′-AGAAGAGTGGTATTTATCGCAGTTGTTTGTAG-TAAAAGA-3′
	36C8R	5′-TCTTTTACTACAAACAACTGCGATAAATACCAC-TCTTCT-3′
